# Physical activity in the morning and afternoon is lower in patients with chronic obstructive pulmonary disease with morning symptoms

**DOI:** 10.1186/s12931-018-0749-4

**Published:** 2018-03-27

**Authors:** Amanda R. van Buul, Marise J. Kasteleyn, Niels H. Chavannes, Christian Taube

**Affiliations:** 1 Department of Pulmonology, Leiden University Medical Center, Albinusdreef 2, Postzone C2-R, Postbus 9600, 2300 RC Leiden, The Netherlands; 2 Department of Public Health and Primary Care, Leiden University Medical Center, Albinusdreef 2, postzone V-0p, Postbus 9600, 2300 RC Leiden, The Netherlands; 3 Department of Pulmonary Medicine, Ruhrlandklinik, West German Lung Center, University Hospital Essen, University Duisburg-Essen, Tüschener Weg 40, 45239 Essen, Germany

**Keywords:** Afternoon, Chronic obstructive pulmonary disease, Evening, Physical activity, Morning, Morning symptoms

## Abstract

**Background:**

Patients with chronic obstructive pulmonary disease (COPD) experience symptoms that vary over the day. Symptoms at the start of the day might influence physical activity during the rest of the day. Therefore, physical activity during the course of the day was studied in patients with low and high morning symptom scores.

**Methods:**

This cross-sectional observational study included patients with moderate to very severe COPD. Morning symptoms were evaluated with the PRO-morning COPD Symptoms Questionnaire (range 0–60); the median score was used to create two groups (low and high morning symptom scores). Physical activity was examined with an accelerometer. Activity parameters during the night, morning, afternoon and evening were compared between patients with low and high morning symptom scores using independent t-tests or Mann-Whitney U tests.

**Results:**

Seventy nine patients were included. Patients were aged (mean ± SD) 65.6 ± 8.8 years with a mean forced expiratory volume in 1 s of 55 ± 17%predicted. Patients with low morning symptom scores (score < 17.0) took more steps in the afternoon (*p* = 0.015) and morning (*p* = 0.030). There were no significant differences during the evening and night.

**Conclusion:**

Patients with high morning symptom scores took significantly fewer steps in the morning and afternoon than those with low morning symptom scores. Prospective studies are needed to prove causality between morning symptoms and physical activity during different parts of the day.

**Electronic supplementary material:**

The online version of this article (10.1186/s12931-018-0749-4) contains supplementary material, which is available to authorized users.

## Background

Chronic obstructive pulmonary disease (COPD) is a worldwide problem with a high prevalence of years lived with disability [1]. COPD is associated with dyspnoea, fatigue and decreased physical activity. Regular physical activity in patients with COPD is associated with a lower risk of admissions and mortality [2]. Most previous studies reported on physical activity during day time solely [3–6]. When physical activity was measured over the course of the day, nuanced reporting on physical activity during different parts of the day was generally lacking [7–10]. Therefore, only little is known about physical activity in COPD patients during the night and early morning. Studying physical activity during the course of the day is important, because a symptomatic start to the day might influence physical activity during the rest of the day.

Patients with COPD experience the morning as most symptomatic part of the day [11, 12] and the night as second most symptomatic part of the day [12]. Studies have shown that morning symptoms are negatively associated with self-reported physical activity [13]. A negative association between morning symptoms and overall physical activity has been reported [14]. However, the relation between morning symptoms and objectively measured physical activity during different parts of the day has not been studied yet. From previous research it is known that morning symptoms influence self-care, housework and work activities [15]. We hypothesized that patients with high morning symptom scores are less active in the morning and during the rest of the day compared to patients with low morning symptom scores. Furthermore, detailed assessments of physical activity over the day could give insight which part might be most suitable for physical activity interventions. Therefore, the primary aim of this study was to examine physical activity during the course of the day depending on morning symptoms. In addition, self-reported physical activity was assessed to better understand which types of activities were generally undertaken.

## Methods

### Research design

The Morning symptoms in-Depth observAtional Study (MODAS) was a single centre, observational, cross-sectional study (study NL51951.058.15; www.toetsingonline.nl). The study was conducted from September 2015 to February 2017. The medical ethics committee from the Leiden University Medical Center (LUMC) approved the study protocol.

### Participants

Detailed inclusion/exclusion criteria have been reported previously [14]. In summary, included in the study were patients aged 40 to 80 years. A physician had diagnosed them with COPD. They had moderate to very severe airflow limitation according to the Global initiative for Obstructive Lung Disease (GOLD) definitions [16]. Patients were exacerbation-free for at least 2 months. They were current or active smokers with at least 10 pack-years. Main exclusion criteria were the diagnosis of asthma; significant other lung disease, comorbidities and severe pain syndromes that impair exercise capacity. Patients were recruited from the LUMC and Alrijne hospital in Leiden (The Netherlands). Patients who were considered to be eligible were notified about the study by telephone or during an outpatient visit. Furthermore, patients were recruited by flyers in local papers. Eligible interested patients received an information letter. If the patient agreed to participate in the study, a study visit was scheduled. During the visit, written informed consent was obtained.

### Assessments

During the baseline visit, demographic data and comorbidities were obtained by a physician. Patients were asked about their medication use and this was verified with a medication overview of the pharmacy. COPD-specific health-related quality-of-life, morning symptoms severity, self-reported physical activity and pre- and post-bronchodilator pulmonary function were assessed at the study center. After this visit, patients wore a triaxial accelerometer for seven consecutive days to objectively assess physical activity. When the physical activity measurements were finished, there was a follow-up telephone interview to report possible adverse events.

Morning symptom severity was evaluated with the PRO-Morning COPD Symptoms Questionnaire (pre-morning doses assessment) [17]. This questionnaire consists of six questions about dyspnoea, cough, sputum production, wheezing, chest tightness and limitations in the morning. Patients rated the severity of these symptoms with a Likert scale from 0 to 10 points. 0 for no symptoms; 10 points for symptoms as bad as they can imagine. The total score ranged from 0 to 60.

Physical activity was objectively measured with an accelerometer (Dynaport MoveMonitor, McRoberts BV, The Hague, the Netherlands) [18, 19]. This accelerometer was worn on the waist during the entire day for seven consecutive days resulting in real-life activity recording. To give insight into physical activity over the course of the day, days were divided in four parts of the day of each 6 hours: night (00.00 to 06.00), morning (06.00 to 12.00), afternoon (12.00 to 18.00) and evening (18.00 to 00.00). Duration of activity (standing, shuffling and walking) and inactivity (sitting and lying) in minutes was registered. The number of steps was recorded per part of the day and per hour. Patients were not allowed to wear the accelerometer during bathing or showering. The duration that the accelerometer was not worn was automatically registered. A measurement was considered valid when patients wore the accelerometer at least 90% per part of the day. Averages of the outcomes of valid parts of the day were calculated for each patient. Patients with only invalid parts of the day were excluded from analysis.

Patients filled out the Dutch version of the international physically activity questionnaire (IPAQ) [20]. In this questionnaire patients reported the number of minutes per day and days per week they were physical active in a 7-day period. Physical activity was categorized in four domains: work, transport, housework or leisure time related. Patients who reported more than 960 min a day each day of the week were excluded from this analysis in line with the activity calculation instructions of the IPAQ.

The Charlson Co-morbidity index was used to evaluate comorbidities (CCI) [21] and the three most common comorbidities were reported as percentage. COPD-specific health-related quality-of-life was assessed with the St George’s Respiratory Questionnaire (SGRQ), [22] health status with the Clinical COPD Questionnaire (CCQ) [23] and dyspnoea in daily living with the medical modified research council (mMRC) [24]. All patients performed pre- and post-bronchodilator spirometry following ERS/ATS (European Respiratory Society/American Thoracic Society) standards in the morning between 08.30 and 11.00 (Care Fusion, Masterscreen PFT). [25–27] In this study, reported spirometry outcomes were post-bronchodilator values. Forced volume in 1 second (FEV_1_) was expressed in % predicted values (based on the Global Lung Function Initiative 2012) [26].

### Statistical analysis

Descriptive data were reported as percentages, mean values ± standard deviations (SD) for normal distributed continuous variables and median with interquartile ranges (IQR) for non-normal distributed continuous variables. The study cohort was divided in two equal groups using the median score on the PRO-morning COPD Symptoms Questionnaire as cut-off. Differences in baseline characteristics between the two groups were compared with an independent t-test for continuous normal distributed variables; a Mann-Whitney U test for continuous non-normal distributed variables and chi-square test for categorical variables.

Differences in steps during the total day were compared between patients with low and high morning symptom scores using an independent t-test. Difference in steps and duration of (in)activity during different parts of the day were compared using an independent t-test or Mann-Whitney U test. To give an overview of the number of steps over the course of the day, steps per hour were plotted against hour of the day. Self-reported physical activity was compared using a Mann-Whitney U test. An explorative subgroup analysis was performed to examine long-acting pulmonary medication use. Long-acting pulmonary medication use was categorized in three groups: double bronchodilation (combination of a long-acting beta2 agonist (LABA) and long-acting muscarinic antagonist (LAMA)), a single bronchodilator (LABA or LAMA use) or no use of long-acting pulmonary medication. The difference in mean number of steps in the morning was evaluated with a one way ANOVA.

Steps per hour were plotted against hour of the day for patients with low and high dyspnoea scores in daily living [16] to evaluate the impact of using a general symptom questionnaire instead of a specific morning symptom questionnaire to divide the study group.

A sensitivity analysis was performed to evaluate the impact of adverse events during the study period; all patients who reported an adverse events, defined as self-reported illness or any somatic symptom for which the patient had to visit a health care provider, were excluded from the analyses.

For all analyses, a *p*-value of < 0.05 was considered statistically significant. Missing data was not replaced. We used SPSS version 23 to perform the analyses.

## Results

### Patients

**Fig. 1:**
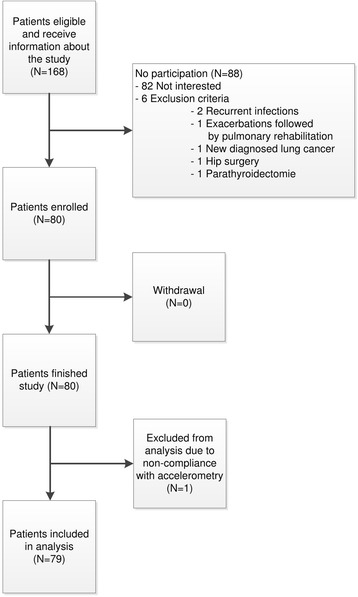
Study flow diagram

From 168 eligible patients who received the patient information form, 80 patients were included in the study and 79 patients had sufficient outcomes from accelerometry for analyses (Fig. 1). Table 1 shows demographics and baseline characteristics. Patients were (mean ± SD) 65.6 ± 8.8 years old and 53% were male. They had a mean FEV_1_ of 55 ± 17% predicted. Most patients were classified as COPD GOLD D (40.5%) and B (27.8%). Mean morning symptom score was 17.9 ± 11.8 with a range between 0 and 47**.** The median morning symptom score was 17.0 and was used as cut-off to separate the study cohort in two equal groups (Table 1).

**Table 1:** Baseline characteristics

Characteristic	All included patients (*N* = 79)	Morning symptom score < 17.0 (*N* = 41)	Morning symptom score ≥ 17.0 (*N* = 38)	Difference (*P*-value)
Age in years, mean (SD)	65.6 (8.8)	66.4 (8.2)	64.7 (9.4)	0.38
Male, n (%)	42 (53)	25 (61)	17 (45)	0.15
Ethnicity Caucasian, n (%)	78 (99)	40 (98)	38 (100)	0.33
Current smoking, n (%)	21 (27)	8 (20)	13 (34)	0.14
Pack years, median [IQR]	37 [25–51]	34 [25–42]	41 [26–71]	0.06
In current employment, n (%)	21 (27)	12 (29)	9 (24)	0.58
BMI in kg/m^2^, mean (SD)	26.4 (5.1)	25.5 (4.8)	27.3 (5.3)	0.13
FEV_1_/FVC ratio, mean (SD)	45.5 (12.2)	45.6 (13.6)	45.5 (10.6)	0.99
FEV_1_% predicted, mean (SD)	55.2 (16.9)	57.2 (19.3)	53.0 (14.0)	0.28
Exacerbation in the previous year, n (%)	41 (52)	16 (39)	25 (66)	0.017
GOLD stage				
A, n (%)	19 (24.1)	17 (41.5)	2 (5.3)	< 0.001
B, n (%)	22 (27.8)	9 (22.0)	13 (34.2)	0.23
C, n (%)	6 (7.6)	6 (14.6)	0 (0.0)	0.014
D, n (%)	32 (40.5)	9 (22.0)	23 (60.5)	< 0.001
CCQ total score, mean (SD)	2.1 (1.1)	1.4 (0.86)	2.8 (0.9)	< 0.001
SGRQ total score, mean (SD)	43.0 (18.6)	32.1 (16.6)	54.8 (12.4)	< 0.001
Long-acting bronchodilation				
Use of one long-acting bronchodilator, n (%)	17 (21.5)	10 (24.4)	7 (18.4)	0.52
Use of two long-acting bronchodilators, n (%))	58 (74.7)	28 (68.3)	31 (81.6)	0.18
No long-acting bronchodilator, n (%)	3 (3.8)	3 (7.3)	0 (0.0)	0.09
CCI score, median [IQR]	2 [1–3]	2 [1–3]	2 [1–3]	0.98
History of solid tumor without metastasis, n (%)	15 (19.0)	9 (22.0)	6 (15.8)	0.49
Cerebrovascular disease, n (%)	10 (12.7)	6 (14.6)	4 (10.5)	0.58
Uncomplicated diabetes mellitus, n (%)	9 (11.4)	3 (7.3)	6 (15.8)	0.24

*BMI* body mass index, *CCI* Charlson comorbidity index, *CCQ* clinical COPD questionnaire, *FEV*_*1*_ Forced expiratory volume in 1 s, *FVC* forced vital capacity, *GOLD* global initiative for chronic obstructive lung disease, *IQR* interquartile range, *SD* standard deviation, *SGRQ* St George Respiratory Questionnaire

### Physical activity

Seventy nine patients wore the accelerometer 7 consecutive days. Thus, data from 553 days were collected from these 79 patients. 95.8% of the night, 91.0% of the morning, 96.7% of the afternoon and 93.9% the evening measurements fulfilled the quality standards and were included in the analysis. In each part of the day, most of the time was spent in inactivity (Table 2).

**Table 2:** Duration of different types of activity during the morning, afternoon, evening and night (N = 79)

Activity	Total duration in mean min (SD)			
	Morning	Afternoon	Evening	Night
Active time^a^	85.5 (39.7)	112.4 (42.3)	57.2 (34.1)	8.8 (10.1)
Standing	54.9 (25.4)	67.8 (25.5)	38.7 (25.9)	6.1 (7.4)
Shuffling	8.7 (5.4)	11.6 (6.7)	5.2 (4.1)	0.8 (1.0)
Walking	22.0 (15.1)	33.0 (20.0)	13.2 (9.8)	1.9 (2.4)
Inactive time^b^	271.6 (39.7)	246.7 (42.0)	302.0 (34.1)	351.1 (10.1)
Lying	150.6 (63.9)	42.9 (45.8)	105.7 (78.5)	327.3 (51.2)
Sitting	120.9 (44.7)	203.8 (45.6)	196.3 (72.4)	23.8 (43.3)
Not worn	2.9 (4.7)	1.0 (1.8)	0.9 (2.2)	0.2 (0.7)

^a^Active: standing, shuffling and walking combined

^b^inactive: lying and sitting combined. SD: standard deviation

**Fig. 2:**
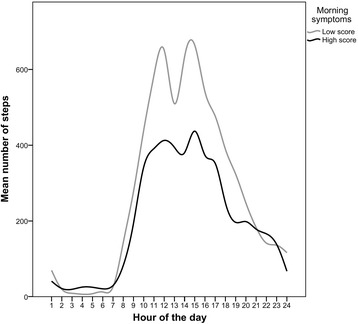
Steps during each hour of the day, Low morning symptom score: score < 17.0; high morning symptom score: score ≥ 17.0

Mean number of steps per day was (mean ± SD) 5686 ± 3514. Patients with low morning symptom scores took 6598 ± 4243 steps a day; those with high morning symptom scores 4727 ± 2209 steps a day (mean difference 1871, *p* = 0.017). Patients with high morning symptom scores took significantly fewer steps during the morning (mean difference 669, *p* = 0.030) and during the afternoon (mean difference 1013, *p* = 0.015) than patients with low morning symptom scores (Table 3). Patients with low morning symptom scores were active for longer during the afternoon (mean difference 19, *p* = 0.040) and spent more minutes walking during the morning (mean difference 8, *p* = 0.020) and afternoon (mean difference 11, *p* = 0.010). There were peaks in mean number of steps at 15:00 (550 steps per hour) and at 12:00 (535 steps per hour) (Fig. 2). When patients with low and high morning symptom scores were categorized on medication use, there was no significant difference in mean number of steps in the morning between the groups (*p* = 0.057) (Additional file 1: Figure S1). When patients were categorized on high and low mMRC score, there was a significant difference in number of steps in the morning, afternoon and evening (Additional file 2: Figure S2).

**Table 3:** Differences in duration of activity during the night, morning, afternoon and evening between COPD patients with low and high morning symptom scores

		Morning symptom score < 17.0 (N = 41)	Morning symptoms score ≥ 17.0 (N = 38)	P-value
Steps	Night	97 [50–173]	92 [50–138]	0.60
	Morning	2117 (1552)	1448 (1069)	0.030
	Afternoon	3196 (2352)	2183 (1025)	0.015
	Evening	1145 (858)	943 (861)	0.30
Active time (in min, mean (SD) or median [IQR])	Night	6 [2–11]	6 [3–10]	0.76
	Morning	91 (39)	79 (40)	0.18
	Afternoon	122 (45)	102 (38)	0.040
	Evening	52 [37–79]	42 [33–77]	0.19
Standing (in min, mean (SD) or median [IQR])	Night	3 [1;7]	4 [2;8]	0.62
	Morning	56 (24)	53 (28)	0.61
	Afternoon	71 (25)	64 (26)	0.23
	Evening	40 (24)	37 (28)	0.59
Shuffling (in min, mean (SD) or median [IQR])	Night	0 [0;1]	1 [0;1]	0.77
	Morning	9 (6)	8 (5)	0.33
	Afternoon	12 (7)	11 (6)	0.44
	Evening	5 [2;8]	4 [2;6]	0.32
Walking (in min, mean (SD) or median [IQR])	Night	13 [7–19]	9 [6–15]	0.11
	Morning	26 (17)	18 (12)	0.020
	Afternoon	38 (24)	27 (12)	0.010
	Evening	15 (10)	12 (10)	0.23
Inactive time (in min, mean (SD) or median [IQR])	Night	354 [349–358]	354 [350–357]	0.88
	Morning	265 (40)	278 (39)	0.14
	Afternoon	238 (44)	257 (37)	0.043
	Evening	299 (33)	305 (36)	0.41
Lying (in min, mean (SD) or median [IQR])	Night	343 [322–353]	343 [330–355]	0.89
	Morning	140 (52)	161 (74)	0.15
	Afternoon	27 [5–50]	34 [12–68]	0.14
	Evening	90 [44–138]	83 [53–151]	0.71
Sitting (in min, mean (SD) or median [IQR])	Night	9 [3–22]	10 [2–22]	0.74
	Morning	125 (38)	117 (51)	0.45
	Afternoon	203 (41)	205 (51)	0.85
	Evening	198 (68)	195 (78)	0.86

Night *N* = 78, morning N = 78, afternoon N = 79, evening N = 78

*COPD* chronic obstructive pulmonary disease, *IQR* interquartile range, *SD* standard deviation

### Self-reported daily activities

Patients’ median [IQR] self-reported physical activity was 715 [319;1448] minutes a week. Patients spent most of their self-reported physical activity during leisure time (Table 4). Patients with low morning symptom scores spent significantly more time in transport and leisure time activities than those with high morning symptom scores. No significant differences were found for work, housework activities and total activity.

**Table 4:** Self-reported daily activities

IPAQ	Total (*N* = 70)^a^	Morning symptom score < 17.0 (*N* = 34)	Morning symptom score ≥ 17.0 (*N* = 36)	Difference (p-value)
Work, in min/week, median [IQR]	0 [0;0]	0 [0;30]	0 [0;0]	0.13
Transport, in min/week, median [IQR]	155 [11–319]	195 [53–514]	68 [0–270]	0.047
Housework, house maintenance and caring for family, in min/week, median [IQR]	120 [0–608]	105 [0–495]	150 [0–698]	0.89
Recreation, sport and leisure time in min/week, median [IQR]	160 [0–420]	293 [15–604]	60 [0–263]	0.017
Total activity min/week, median [IQR]	715 [219–1448]	935 [533–1808]	665 [131–1298]	0.13

^a^9 out of 79 patients were excluded: 6 patients did not fully complete the IPAQ; 3 patients filled out unreasonably high time in physical activity (more than 960 min a day each day of the week)

*IPAQ* international physical activity questionnaire, *IQR* interquartile range

### Sensitivity analysis

Seven patients reported an adverse event. One patient was exhausted due to the study visit, two patients reported pain in their hands, one had pneumonia, one had flu, one had sinusitis and one patient reported a visit to her general practitioner who prescribed a course of antibiotics and prednisone for pulmonary complaints. There were no serious adverse events.

Patients with adverse events have a significant lower quality of life and reported higher morning symptom scores (Additional file 3: Table S1). When excluding the patients with an adverse event from analyses, the cut-off n the PRO-morning COPD Symptoms Questionnaire to divide the cohort in two equal groups decreased to 15.0, since all patients with an adverse event were categorized in the high morning symptom score group. Results were nearly similar (Additional file 3: Table S2 and S3 and Additional file 4: Figure S3) apart from that there was no difference anymore in number of steps in the morning between patients with low and high morning symptom scores.

## Discussion

This study was designed to evaluate physical activity during the course of the day in patients with moderate to severe COPD with low and high morning symptom scores. This is one of the first studies that explored physical activity in more detail during the course of the day using objective methodology. We showed that patients with high morning symptom scores took significantly fewer steps in the morning and afternoon than those with low morning symptom scores. There were no differences in physical activity in the evening and night between these two groups. Patients with high morning symptom scores spent less time in transport and leisure time than patients with low morning symptom scores.

The present study showed that patients with high morning symptom scores took fewer steps during the total day. This is in line with previous studies that have shown that daily symptoms were associated with lower physical activity levels [10]. One study in a primary care setting in men who are aged between 71 and 92 years, of whom 50% had chronic conditions, [28] reported that men were most active in the morning, followed by a substantial decline in steps per hour, with peaks at times 10:00, 14:00 and 22:00. Our study showed that the afternoon is the most active part of the day for patients with COPD with peaks in steps per hour at times 12:00 and 15:00. Thus, patients with COPD have different activity patterns during the course of the day. These findings are in line with another study of activity in COPD patients; this study showed the highest activity levels during the late morning and early afternoon, followed by a decline in activity [5]. The difference in activity patterns between older men and COPD patients could possibly be explained by known activity characteristics that are typical for COPD patients: lower walking speed, [29] walking with increased duration in time between steps, [30] doing activities slower, [12] taking more breaks, [12] performing daily activities in fewer bouts [4] and shorter bouts [4].

When analysing the activity patterns in a more detailed way, the present study showed that patients with high morning symptom scores were less active during the morning and afternoon than patients with low morning symptom scores. Systematic reviews have shown that multiple determinants have impact on physical activity [31] and that morning symptoms are associated with physical activity [13]. The etiology of morning symptoms is unknown. It can be speculated that inactivity in the afternoon in patients with high morning symptom scores could be due to the long-lasting effects of morning symptoms. It could also be true that patients with morning symptoms have more symptoms during the rest of the day [32]. Interestingly, we found no difference in active time during the evening and night between patients with low and high morning symptom scores. We did not expect this, because we expected that morning symptoms would be associated with less physical activity during each part of the day as patients reported in previous studies in which physical activity was not objectively measured [15, 33]. Exploring the assumption that morning symptoms influence physical activity in the morning, but not in the evening, the evening might be a suitable part of the day in which physical activity could be enhanced, especially in those with high morning symptom scores.

In line with previous research, the present study showed that patients spent most of their self-reported physical activity in leisure time [4]. Patients with high morning symptom scores spent less time in transport and leisure time activities than patients with low morning symptom scores. Encouraging physical activity in leisure time might result in more physical activity and can also increase quality of life [34]. A few previous studies have shown that inhaled medication decreased physical activity limitations due to morning symptoms [15, 35, 36]. An explorative analysis in the present study showed no differences in physical activity between no, single or double long-acting bronchodilator use. However, this study was not powered to show differences in medication use and future research regarding this topic is warranted.

A strength of the current study was 24-h a day accelerometry. This resulted in real-life activity recording without missing physical activity during the evening and the night. However, we did not have information regarding the time patients go to bed and woke up. Consequently, patients who got out of bed early in the morning (and were already awake for a couple of hours), were compared with patients who had only just awoken. Previous studies that assessed the association between morning symptoms and physical activity used self-reported questionnaires, while accelerometers are superior in physical activity assessment than questionnaires [13, 37]. Another strength was the inclusion of patients from an academic medical center, a local hospital and patients recruited by flyers in local papers. This resulted in a heterogeneous population that is generalizable to all patients with moderate to very severe COPD. A limitation of the study was that the accelerometer was not waterproof and patients were not allowed to wear it while taking a shower. For some patients, taking a shower is one of the main physical activities during a day, and this activity was not measured. This means, active time was underestimated. A second limitation is that morning symptoms were evaluated with a non-validated questionnaire. However, there is no validated morning symptom questionnaire available yet. Patients filled in the questionnaire at the study center and not at home at the time they woke up. This might have result in recall bias that might cause higher (or lower) total morning symptom scores. The mean morning symptom score was slightly higher than in a previous study that used the PRO-Morning COPD Symptoms Questionnaire too [17]. Therefore, it could be possible that the cut-off point to separate high from low morning symptom scores was too high. However, when patients with an AE were removed and the cut-off point dropped to 15.0, the outcomes were nearly the same. Another limitation is the observational design of the study. Therefore, it is not possible to prove whether morning symptoms are fully responsible for the differences in physical activity or that physical inactivity itself resulted in more symptoms due to muscle depletion and loss of physical condition.

The ERS reported in the physical activity statement in COPD that there are only a few randomised controlled trials that studied effects of treatment on physical activity and that there is a need for additional well-designed trials [29]. Taking the outcomes of this study into account, we suggest for future research to focus on two targets to improve physical activity: first, study the etiology of morning symptoms It would be valuable to measure night time symptoms, the effect of flow-limitation during the night (and the early morning) and the effects of spreading physical activity in relation to morning symptoms. Improvement in morning symptoms consequently might increase the number of steps in the morning and afternoon. Second, develop activity programs that encourage physical activity in the evening in addition to daily physical activities. Physical activity programs can be supported by telecoaching and step counters that provide direct feedback [3].

## Conclusion

This study showed that patients with moderate to very severe COPD were most active in the afternoon. Patients with high morning symptom scores took significantly fewer steps in the morning and afternoon than those with low morning symptom scores. Prospective studies are needed to prove causality between morning symptoms and physical activity during different parts of the day.

## Additional files

Additional file 1: **Figure S1. ** Number of steps in the morning, Error bars present 95% confidence intervals. Low morning symptom score: score < 17.0; high morning symptom score: score ≥ 17.0. (JPEG 192 kb)

Additional file 2: **Figure S2.** Steps during each hour of the day, Low mMRC < 2 (*N* = 27); high mMRC ≥2 (*N* = 52). (JPEG 195 kb)

Additional file 3: **Table S1.** Baseline characteristics for patients with and without an adverse event, **Table S2** Differences in activity during the night, morning, afternoon and evening between patient with low and high morning symptom scores, *N* = 72, **Table S3** Daily physical activity. (DOCX 41 kb)

Additional file 4: **Figure S3.** Steps during the course of the day, Few morning symptoms: morning symptom score < 15; severe morning symptoms: morning symptom score ≥ 15. (JPEG 203 kb)

## Abbreviations

ATS: American thoracic society
BMI: Body mass index
CCI: Charlson Co-morbidity index
CCQ: Clinical COPD questionnaire
COPD: Chronic obstructive pulmonary disease
ERS: European respiratory society
FEV_1_: Forced expiratory volume in 1 s
FVC: Forced vital capacity
GOLD: Global initiative for chronic obstructive lung disease
IPAQ: international physical activity questionnaire
IQR: Interquartile ranges
LABA: Long-acting beta2 agonist
LAMA: Long-acting muscarinic antagonist
LUMC: Leiden University Medical Center
mMRC: Modified medical research council
MODAS: Morning symptoms in-Depth observational study
SD: Standard deviation
SGRQ: St George’s Respiratory Questionnaire
