# Supplementary figures and images for: Physical activity in the morning and afternoon is lower in patients with chronic obstructive pulmonary disease with morning symptoms
Source: Respir Res. 2018 Mar 27;19:49. doi: 10.1186/s12931-018-0749-4 (PMC5870529; doi:10.1186/s12931-018-0749-4)

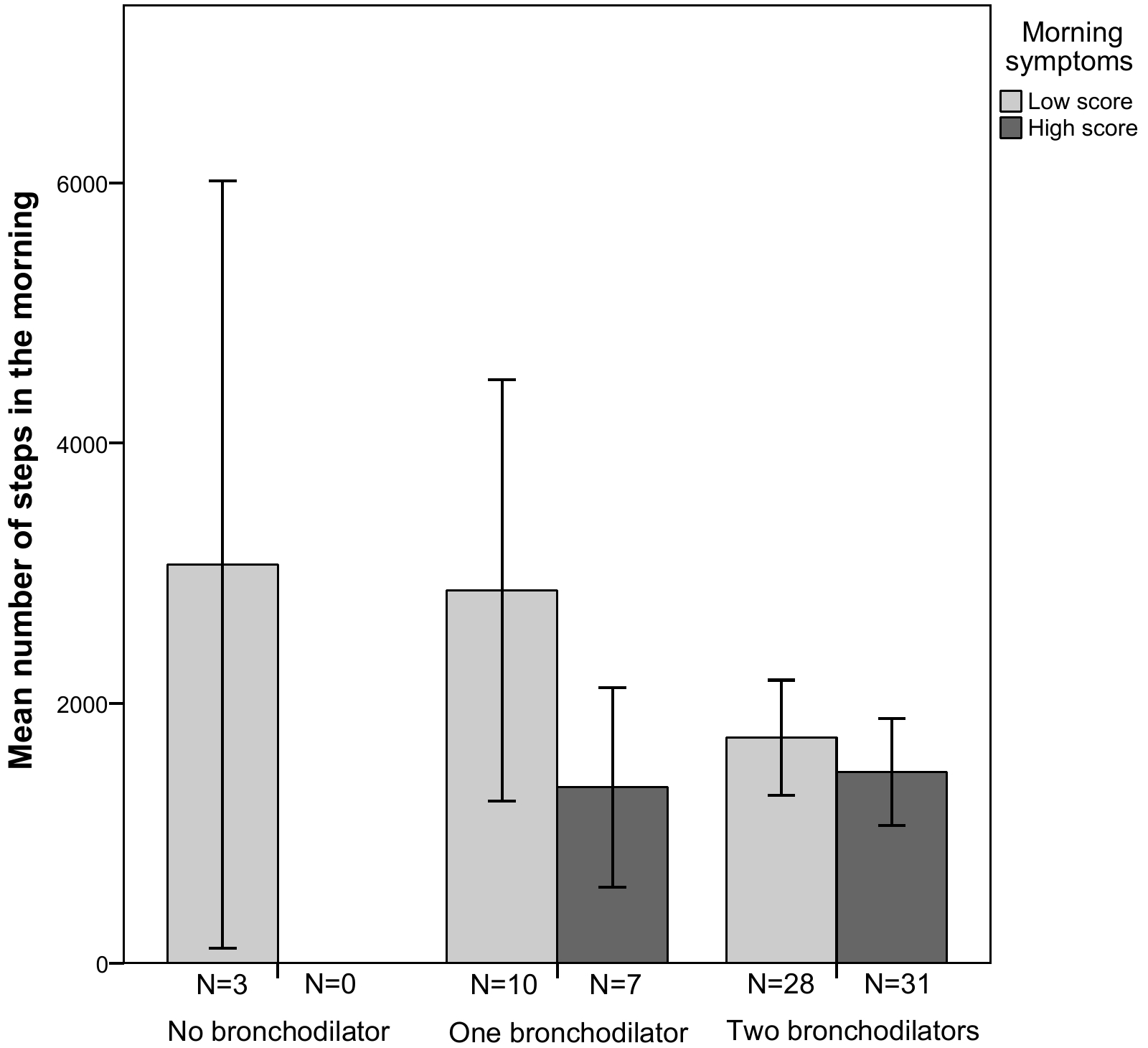

Supplement: Supplementary file 1 — Figure S1. Number of steps in the morning, Error bars present 95% confidence intervals. Low morning symptom score: score < 17.0; high morning symptom score: score ≥ 17.0. (JPEG 192 kb) [file 12931_2018_749_MOESM1_ESM.jpg]

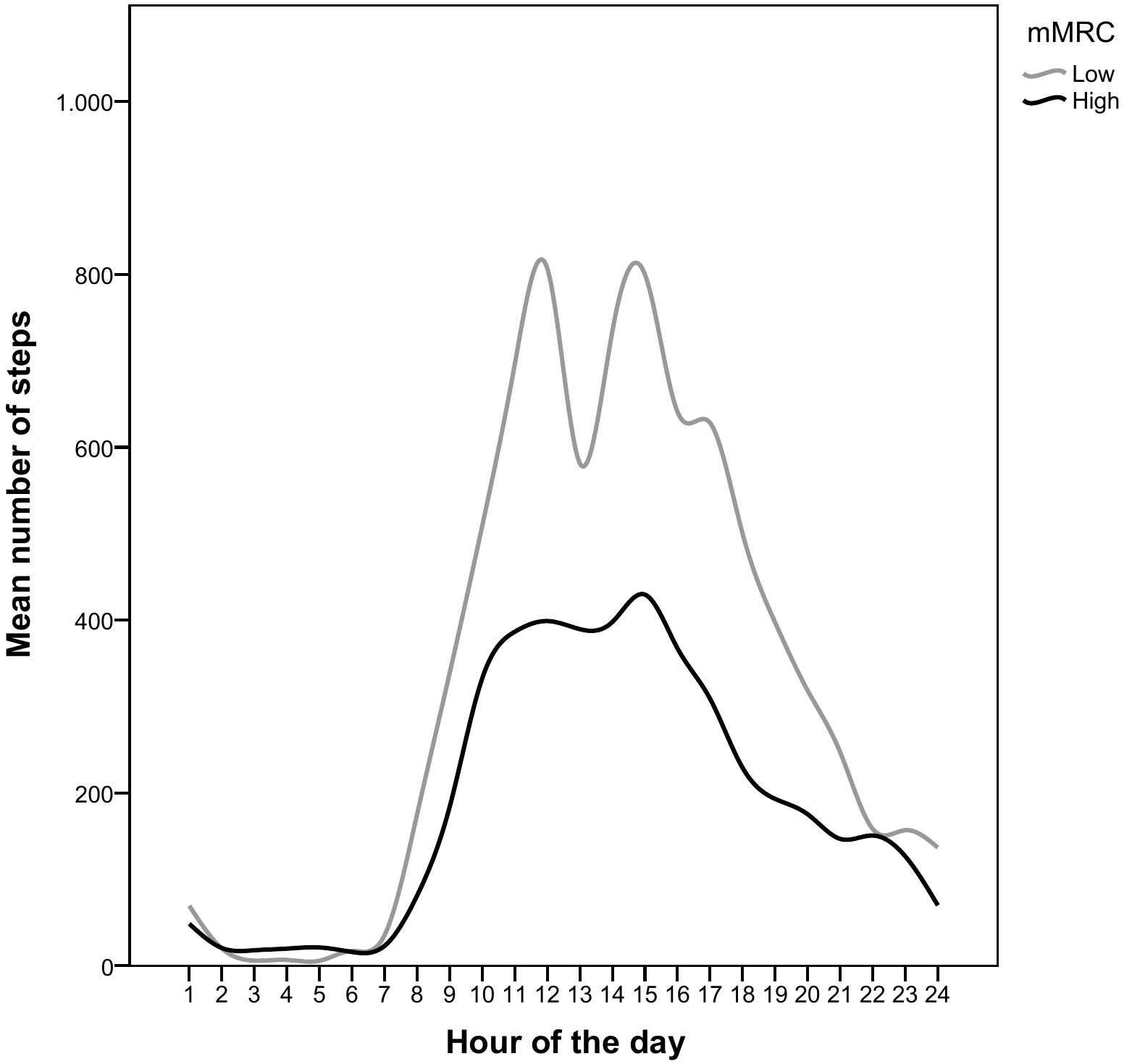

Supplement: Supplementary file 2 — Figure S2. Steps during each hour of the day, Low mMRC < 2 (N = 27); high mMRC ≥2 (N = 52). (JPEG 195 kb) [file 12931_2018_749_MOESM2_ESM.jpg]

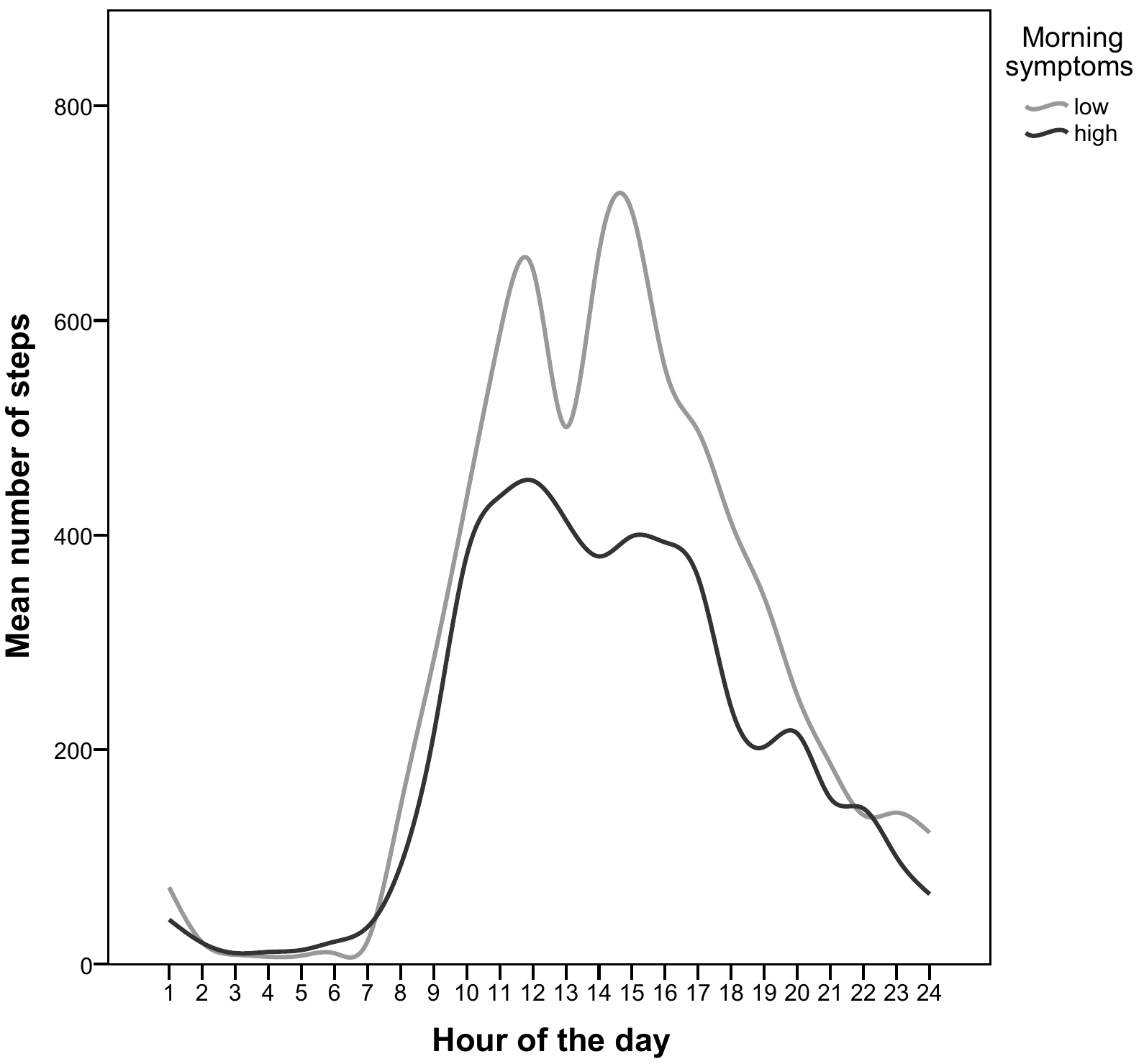

Supplement: Supplementary file 4 — Figure S3. Steps during the course of the day, Few morning symptoms: morning symptom score < 15; severe morning symptoms: morning symptom score ≥ 15. (JPEG 203 kb) [file 12931_2018_749_MOESM4_ESM.jpg]
