# Supplementary material for: Physical activity in the morning and afternoon is lower in patients with chronic obstructive pulmonary disease with morning symptoms
Source: Respir Res. 2018 Mar 27;19:49. doi: 10.1186/s12931-018-0749-4 (PMC5870529; doi:10.1186/s12931-018-0749-4)
Supplement: Supplementary file 3 — Table S1. Baseline characteristics for patients with and without an adverse event, Table S2 Differences in activity during the night, morning, afternoon and evening between patient with low and high morning symptom scores, N = 72, Table S3 Daily physical activity. (DOCX 41 kb) [file 12931_2018_749_MOESM3_ESM.docx]

**Additional file 3 Table S1** Baseline characteristics for patients with and without an adverse event

| Characteristic | | | All included patients (N = 79) | With AE (N=7) | Without AE (N=72) | Difference (p-value) |
| --- | --- | --- | --- | --- | --- | --- |
| Age in years, mean (SD) | | | 65.6 (8.8) | 69.4 (7.2) | 65.2 (8.9) | 0.23 |
| Male, n (%) | | | 42 (53) | 4 (57) | 38 (53) | 0.83 |
| Ethnicity Caucasian, n (%) | | | 78 (99) | 7 (100) | 71 (99) | 0.75 |
| Current smoking, n (%) | | | 21 (27) | 1 (14) | 20 (28) | 0.44 |
| Pack years, mean (SD) | | | 37 [25-51] | 51 [30-110] | 35 [25-50] | 0.10 |
| In current employment, n (%) | | | 21 (27) | 0 (0) | 21 (29) | 0.10 |
| BMI in kg/m^2^, mean (SD) | | | 26.4 (5.1) | 30.5 (8.6) | 26.0 (4.5) | 0.22 |
| FEV1/FVC ratio, mean (SD) | | | 45.5 (12.2) | 47.9 (6.6) | 45.3 (12.6) | 0.60 |
| FEV_1_ % predicted, mean (SD) | | | 55.2 (16.9) | 60.6 (9.5) | 54.6 (17.5) | 0.38 |
| Exacerbation in the previous year, n (%) | | | 41 (52) | 6 (85.7) | 35 (48.6) | **0.06** |
| GOLD stage | | | | | | |
|  | | A, n (%) | 19 (24.1) | 0 (0.0) | 19 (26.4) | 0.12 |
|  | | B, n (%) | 22 (27.8) | 2 (28.6) | 20 (27.8) | 0.96 |
|  | | C, n (%) | 6 (7.6) | 0 (0.0) | 6 (8.3) | 0.43 |
|  | | D, n (%) | 32 (40.5) | 5 (71.4) | 27 (37.5) | 0.08 |
| CCQ total score, mean (SD) | | | 2.1 (1.1) | 3.0 (0.7) | 2.0 (1.1) | **0.029** |
| SGRQ total score, mean (SD) | | | 43.0 (18.6) | 59.1 (11.5) | 41.4 (18.4) | **0.015** |
| Long-acting bronchodilation | | | | | | |
|  | Use of one long-acting bronchodilator, n (%) | | 17 (21.5) | 1 (14.3) | 16 (22.2) | 0.63 |
|  | Use of two long-acting bronchodilators, n (%)) | | 59 (74.7) | 6 (85.7) | 53 (73.6) | 0.48 |
|  | No long-acting bronchodilator, n (%) | | 3 (3.9) | 0 (0.0) | 3 (4.2) | 0.58 |
| CCI score, median [IQR] | | | 2 [1;3] | 2 [1;2] | 2 [1;3] | 0.91 |
|  | | History of solid tumor without metastasis, n (%) | 15 (19.0) | 0 (0.0) | 15 (20.8) | 0.18 |
|  | | Cerebrovascular disease, n (%) | 10 (12.7) | 0 (0.0) | 10 (13.9) | 0.29 |
|  | | Uncomplicated diabetes mellitus, n (%) | 9 (11.4) | 3 (42.9) | 6 (8.3) | **0.006** |
| Morning symptom score, mean (SD) | | | 17.9 (11.8) | 29.3 (5.7) | 16.8 (11.7) | **<0.001** |

AE: adverse event; BMI: body mass index; CCQ: clinical COPD questionnaire; CCI: Charlson co-morbidity index; FEV_1_: Forced expiratory volume in 1 second; FVC: forced vital capacity; IQR: interquartile range; SD: standard deviation, SGRQ: St George Respiratory Questionnaire

**Additional file 3 Table S2 Differences in activity during the night, morning, afternoon and evening between patient with low and high morning symptom scores, N=72**

|  | | Few morning symptoms (score <15) | Severe morning symptoms (score ≥15) | Difference (p-value) |
| --- | --- | --- | --- | --- |
| Steps | Night | 89 [50;181] | 91 [41;143] | 0.47 |
|  | Morning | 2122 (1624) | 1609 (1071) | 0.12 |
|  | Afternoon | 3309 (2449) | 2186 (990) | **0.013** |
|  | Evening | 1182 (893) | 883 (652) | 0.11 |
| Active time (in min, mean (SD) or median [IQR]) | Night | 6 [2;11] | 5 [3;11] | 0.85 |
|  | Morning | 92 (39) | 85 (39) | 0.44 |
|  | Afternoon | 125 (45) | 103 (37) | **0.023** |
|  | Evening | 55 [34;80] | 46 [33;76] | 0.21 |
| Standing (in min, mean (SD) or median [IQR]) | Night | 3 [1;8] | 3 [2;7] | 0.66 |
|  | Morning | 57 (23) | 57 (28) | 0.97 |
|  | Afternoon | 72 (24) | 65 (26) | 0.21 |
|  | Evening | 42 (25) | 39 (29) | 0.63 |
| Shuffling (in min, mean (SD) or median [IQR]) | Night | 0 [0;1] | 0 [0;1] | 0.80 |
|  | Morning | 9 (6) | 8 (5) | 0.37 |
|  | Afternoon | 13 (7) | 10 (5) | 0.11 |
|  | Evening | 5 [2;8] | 4 [2;6] | 0.31 |
| Walking (in min, mean (SD) or median [IQR]) | Night | 13 [7;20] | 10 [6;15] | 0.15 |
|  | Morning | 26 (17) | 20 (12) | 0.11 |
|  | Afternoon | 40 (25) | 27 (11) | **0.008** |
|  | Evening | 15 (10) | 11 (8) | 0.11 |
| Inactive time (in min, mean (SD) or median [IQR]) | Night | 354 [349;358] | 354 [349;357] | 0.88 |
|  | Morning | 264 (40) | 273 (38) | 0.36 |
|  | Afternoon | 234 (45) | 256 (37) | **0.023** |
|  | Evening | 297 (34) | 304 (36) | 0.40 |
| Lying (in min, mean (SD) or median [IQR]) | Night | 343 [317;353] | 342 [331;355] | 0.75 |
|  | Morning | 140 (53) | 154 (68) | 0.31 |
|  | Afternoon | 27 [7;48] | 44 [12;84] | 0.08 |
|  | Evening | 79 [43;134] | 100 [51;196] | 0.35 |
| Sitting (in min, mean (SD) or median [IQR]) | Night | 9 [3;31] | 10 [2;16] | 0.54 |
|  | Morning | 125 (40) | 119 (46) | 0.56 |
|  | Afternoon | 200 (40) | 200 (49) | 0.95 |
|  | Evening | 200 (66) | 187 (81) | 0.45 |

Night N=71, morning N=71, afternoon N=72, evening N=71. IQR: interquartile range; SD: standard deviation.

Additional file 3 Table S3 Daily physical activity

| IPAQ | Total (N=64)* | A few morning symptoms (N=31) | Severe morning symptoms (N=33) | Difference (p-value) |
| --- | --- | --- | --- | --- |
| Work, in min/week, median [IQR] | 0 [0;0] | 0 [0;30] | 0 [0;0] | 0.31 |
| Transport, in min/week, median [IQR] | 155 [5;326] | 210 [30;525] | 60 [0;240] | **0.015** |
| Housework, house maintenance and caring for family, in min/week, median [IQR] | 120 [0;653] | 180 [0;540] | 120 [0;780] | 0.67 |
| Recreation, sport and leisure time in min/week, median [IQR] | 180 [0;465] | 345 [20;615] | 60 [0;285] | **0.035** |
| Total activity min/week, median [IQR] | 815 [338;1478] | 990 [600;1920] | 540 [90;1285] | **0.047** |

*5 patients did not fully complete the IPAQ; 3 patients filled out unreasonably high time in physical activity (more than 960 minutes a day each day of the week). IPAQ: international physical activity questionnaire; IQR: interquartile range
